# Negotiating decisions on aggressive cancer care at end-of-life between patients, family members, and physicians – A qualitative interview study

**DOI:** 10.3389/fonc.2022.870431

**Published:** 2022-09-23

**Authors:** Markus W. Haun, Alina Wildenauer, Mechthild Hartmann, Caroline Bleyel, Nikolaus Becker, Dirk Jäger, Hans-Christoph Friederich, Justus Tönnies

**Affiliations:** ^1^ Department of General Internal Medicine and Psychosomatics, Heidelberg University, Heidelberg, Germany; ^2^ Department of Child and Adolescent Psychiatry, Heidelberg University, Heidelberg, Germany; ^3^ Division of Cancer Epidemiology, German Cancer Research Center (DKFZ), Heidelberg, Germany; ^4^ Department of Medical Oncology, National Center for Tumor Diseases (NCT), Heidelberg, Germany

**Keywords:** cancer, aggressiveness of care, decision regret, decision-making, end-of-life, chemotherapy, qualitative interview

## Abstract

**Background:**

Patients with advanced cancer do receive increasingly aggressive end-of-life care, despite it does often not prolong survival time but entails decreased quality of life for patients. This qualitative study explores the unfolding of aggressive end-of-life care in clinical practice focusing on the decision-making process and the quality of end-of-life care from family members’ perspective.

**Materials and methods:**

We conducted semi-structured interviews with 16 family members (six of cancer patients with and ten without aggressive end-of-life care) at the National Center for Tumor Diseases Heidelberg, Germany. We conducted a content analysis applying a theoretical framework to differentiate between ‘decision-making’ (process of deciding for one choice among many options) and ‘decision-taking’ (acting upon this choice).

**Results:**

While patients of the aggressive care group tended to make and take decisions with their family members and physicians, patients of the other group took the decision against more aggressive treatment alone. Main reason for the decision in favor of aggressive care was the wish to spend more time with loved ones. Patients took decisions against aggressive care given the rapid decline in physical health and to spare relatives difficult decisions and arising feelings of guilt and self-reproach.

**Conclusion:**

Treatment decisions at end-of-life are always individual. Nevertheless, treatment courses with aggressive end-of-life care and those without differ markedly. To account for a longitudinal perspective on the interplay between patients, family members, and physicians, cohort studies are needed. Meanwhile, clinicians should validate patients and family members considering refraining from aggressive end-of-life care and explore their motives.

**Clinical trial registration:**

https://www.drks.de/drks_web/navigate.do?navigationId=trial.HTML&TRIAL_ID=DRKS00022837, identifier DRKS00022837.

## Background

In recent years, end-of-life treatment of advanced-stage cancer patients has become increasingly intensive and prolonged, as demonstrated for the United States, Canada and several European countries (1–5). To define specific indicators for the intensive treatment, the term ‘aggressive end-of-life cancer care’ has been introduced. By definition, ‘aggressive end-of-life cancer care’ is present if one or more of the following criteria are present: 1) a new chemotherapy regimen starting less than 30 days before death, 2) the last dose of chemotherapy within 14 days of death, 3) more than one emergency visit in the last month of life, 4) more than 14 days in hospital in the last month of life, 5) more than one hospital admission in the last month of life, or 6) an ICU admission in the last month of life (6).

Although the increased use of aggressive end-of-life care is usually driven by a desire to provide the best medical care, studies show that it is often not associated with longer survival or a better quality of life for patients (7–9). To understand why cancer patients nevertheless receive that kind of treatment, very few studies have investigated the involvement and perspectives of the various stakeholder groups involved in the negotiation of end-of-life care, such as oncologists, nurses, primary physicians, family members, and patients themselves (10–13). Patients seem to consider cancer treatment to be generally useful and seem to choose to undergo such treatment even if their chances of survival gain are little improved (14, 15). Physicians reinforce these attitudes of “not giving up” by emphasizing the continuation of treatment, e.g. by offering or escalating chemotherapy (10). While patients’ and clinicians’ attitudes towards and contributions to aggressive end-of-life care have been studied, family members’ perspectives remain largely unknown.

To capture the relevant evidence on this stakeholder group systematically, we searched MEDLINE from January 1st, 1946, until February 6th, 2022, without language or date restriction, for qualitative studies on family members of advanced cancer patients’ and their perspective on and involvement in aggressive end-of-life cancer care. In **Supplemental Appendix 1**, we present the detailed search terms. We found eleven qualitative studies, which focused on the experiences of family members of cancer patients with end-of-life cancer care in general, which included other aspects, e.g., palliative care. Only two studies focused on the family member’s perspective on the use of *aggressive* end-of-life cancer care. Family members reported less quality of care, when aggressive end-of-life care had been initiated (12). Nevertheless, family members tend to respect the wish for continuation of treatment of their family members, although it might contrast from their own opinion concerning the use of aggressive care (16).

To understand and further investigate the process of decision-making, it is important to consider the work of Herbert A. Simon on decision-making. His theory of behavioral and cognitive processes of humans making rational decisions postulates two crucial steps – the “process of decision as well as [with] the process of action” (17). Specifically, Simon differentiates between 1) the process of actively deciding for one choice among many options and 2) the process of acting upon this choice. In this study, we refer to these two steps as “decision-making” and “decision-taking”. According to Simon, during the decision-process, individuals are affected by limited knowledge and various psychological, environmental, and social factors. Thus, Simon generally rejects the assumption of perfect rationality.

When it comes to decision-processes, there are few more important decisions than how to deal with a potentially life-threatening malignant disease. Patients, family members, and physicians usually must tackle with this challenge on a day-to-day basis. Given the scarcity of data on the family members’ perspective and the decision-making process, the main aim of our study was to give a thorough understanding of the involvement of family members of advanced-stage cancer patients. Therefore, our research focused on (1) identifying the extent and nature of family members’ participation in the decision-making process, (2) investigating potential decision-making differences between the group of family members who experienced aggressive end-of-life care in the patients and the group of family members who did not, and (3) delineating motives for decisions against aggressive end-of-life care.

## Methods

### Study design

This study reports findings from the qualitative strand of a larger mixed-methods research project (“Determining factors and implications of aggressiveness of care towards the end of life of cancer patients from a caregiver’s perspective”) exploring decision-making in the treatment trajectory of patients with advanced cancer, who had received their treatment at the National Center for Tumor Diseases (NCT) in Heidelberg, a large, tertiary comprehensive cancer care center in Germany, from the perspective of their bereaved family members. The project was registered in the German Clinical Trials Register (registration number: DRKS00022837). The findings from the quantitative strand are published elsewhere (18). In the qualitative strand featuring one-off semi structured interviews with bereaved family members, we took a critical realist stance to collecting and analyzing the data. That is, while assuming social structures independent of our understanding (e.g., general imperative for patients to participate in treatment), we examined how participants constructed meanings when engaging with these structures (e.g., how does the individual patient deal with his imperative) and in doing so aimed to account for our own experience and background as researchers (e.g., how do clinicians perpetuate this imperative).

### Participants and recruitment

In the first step, bereaved family members of patients with advanced cancer who were listed in the Cancer Registry of the NCT Heidelberg were screened for eligibility for a survey on the frequency of aggressive end-of-life care and potentially related distress (quantitative strand). Family members were eligible if (1) they were named as a contact person by the patients and their contact address could be detected, (2) the patient had had the following types of cancer: pancreatic, prostate, colon, breast, and lung cancer, (3) the patient had died between June 30^th^, 2012, and December 31^st^, 2014, (4) they were aged 18 years or older, and (5) they were proficient in German. Overall, 649 of all 1141 identified family members were eligible. Exclusion criteria were poor German language proficiency, lack of informed consent, and cognitive incapability to respond to interview questions. Eventually, 301 family members responded to the initial survey (response rate: 46.4%). In the second step, at the end of the survey, respondents indicated whether they would be willing to participate in semi structured in-person interviews (qualitative strand). One-hundred-twenty-five family members (41.5%) were interested.

We applied stratified sampling following a purposive strategy considering family member’s age and the item ‘aggressive vs. non- aggressive treatment regime at the end of life’. Specifically, we decided to contrast the experiences by comparing family members from patients who had aggressive end-of-life care in a more “strict sense” as indicated in the survey (criterion: either new chemotherapy regimen starting less than 30 days before death, the last dose of chemotherapy within 14 days of death, or ICU admission in the last month of life) with those family members who had not experienced such treatment in the now deceased. Eventually, we conducted six interviews with family members having experienced aggressive end-of-life care and with ten family members who had not. To ensure a balanced representation between older and younger people, we aimed for a 1:1 recruitment ratio of people aged 18 to 49 years to people aged 50 years or older. Eventually, we recruited 16 interviewees.

### Data collection

To capture participants’ descriptions systematically, we formed an expert panel (working group within the Division of Psycho-Oncology at the Department of General Internal Medicine and Psychosomatics, Heidelberg University) for compiling a semi structured question interview guide consisting of nineteen questions (**Supplemental Appendix 2**). The questions focused on (1) the chronological course of the malignant disease (including the therapy regimen in the last 6 months before death), (2) how decisions on aggressive cancer care at end-of-life were negotiated between patients, and (3) the resulting emotional experience for family members. The proposed guide was pilot tested with two participants and then reviewed. The interviews took place either in the participant’s home or at Heidelberg University Hospital, according to the interviewee’s preference. The interviews were conducted by a trained member of the research group with a master’s degree in Developmental and Clinical Psychology, who had no contact or relationship with any participant prior to the study. The study objectives and data protection guidelines were introduced to the participants. We guaranteed the absence of nonparticipants during the interview. All interviews were audio recorded. Over the course of the entire data collection, we discussed the progress of sampling and data collection focusing on the sample composition and the level of data saturation. We did not repeat any interviews.

### Data analysis

Following the verbatim audio transcription of the recordings by a professional transcription service, we anonymized the data. We did not return the transcripts to the participants for comments. To identify overarching aspects pertaining to the negotiation of decisions on aggressive cancer care at end-of-life between patients, family members, and physicians, two coders (AW and MWH) independently conducted a computer-assisted thematic analysis of two interviews (one aggressive care and one non-aggressive care interview each) in MAXQDA Analytics Pro 2020 (Release 20.1.1, VERBI GmbH) (19). At this point, the coders developed the coding system following a combined bottom-up (data-driven) and top-down strategy (20). For the latter, we applied the distinction between “decision-making” and “decision-taking” according to Simon (17) as an analytical lens to identify differences in the decision processes regarding the involvement of different stakeholders. We discussed disagreements throughout the process in our multidisciplinary research team (sociologist, epidemiologist, clinical psychologist, and medical doctor), until we reached a consensus on a preliminary code system. Both coders then used this preliminary coding system to analyze the remaining 14 transcripts top-down, discussed emerging new themes and modified the codes when necessary. Again, ambiguities arising when coding the data were resolved through discussion in the research team involving MH and JT. We considered theme saturation to be reached when the data did not offer any new themes, that is, when the developed themes represented all the data in the final code system (21). In **Supplement Appendix 3**, we present a summary of the key themes including definitions and supporting quotes. All materials were translated from German to English for this paper by AW and MWH. For reporting our study, we followed the Standards for Reporting Qualitative Research (SRQR) (22).

### Compliance with ethical standards

This study was approved by the Ethics Committee of the Medical Faculty at Heidelberg University (S-500/2014). All procedures performed in studies involving human participants were in accordance with the ethical standards of the institutional and/or national research committee and with the 1964 Helsinki declaration and its later amendments or comparable ethical standards. We obtained written informed consent from all individual participants prior to study enrollment.

## Results

### Sample

In sum, we conducted individual telephone interviews (range 45-80 minutes, median 65 minutes) with 16 bereaved family members consenting to study participation. **Table 1** shows the sociodemographic characteristics. Interviews were conducted between late 2015 and mid-2016.

**Table 1:** Characteristics of interviewees stratified by occurrence of aggressive end-of-life care during the patient’s treatment course.

	aggressive end-of-life care absent (N=10)	aggressive end-of-life care present (N=6)	Overall (N=16)
**Age**			
Mean (SD)	63.9 (13.3)	40.0 (17.2)	54.3 (18.8)
Median [Min, Max]	59.0 [45.0, 80.0]	38.0 [21.0, 70.0]	58.0 [21.0, 80.0]
Missing	1 (10.0%)	0 (0%)	1 (6.2%)
**Gender**			
female	6 (60.0%)	5 (83.3%)	11 (68.8%)
male	4 (40.0%)	1 (16.7%)	5 (31.2%)
**Marital status (at study participation)**			
single	8 (80.0%)	4 (66.7%)	12 (75.0%)
in partnership	1 (10.0%)	2 (33.3%)	3 (18.8%)
Missing	1 (10.0%)	0 (0%)	1 (6.2%)
**Education level (at study participation)**			
≤ 9 years	1 (10%)	0 (0%)	1 (6.3%)
≥ 9 years	8 (80%)	6 (100%)	14 (8%)
Missing	1 (10.0%)	0 (0%)	1 (6.3%)
**I am the … of the deceased**			
partner	9 (90.0%)	2 (33.3%)	11 (68.8%)
child	1 (10.0%)	4 (66.7%)	5 (31.2%)
**Age of deceased in years**			
Mean (SD)	65.1 (13.0)	59.2 (17.0)	62.9 (14.4)
Median [Min, Max]	71.0 [40.0, 77.0]	58.5 [37.0, 79.0]	68.0 [37.0, 79.0]
**Gender of deceased**			
female	5 (50.0%)	2 (33.3%)	7 (43.8%)
male	5 (50.0%)	4 (66.7%)	9 (56.3%)
**Time between initial tumor diagnosis and death of deceased in months**			
Mean (SD)	51.1 (57.7)	32.3 (48.4)	43.6 (53.2)
Median [Min, Max]	41.0 [2.00, 160]	15.0 [1.00, 129]	20.0 [1.00, 160]
Missing	1 (10.0%)	0 (0%)	1 (6.2%)
**Time between death of deceased and study participation in months**			
Mean (SD)	21.6 (13.2)	32.0 (8.32)	25.5 (12.4)
Median [Min, Max]	20.5 [5.00, 49.0]	35.0 [17.0, 39.0]	27.0 [5.00, 49.0]
**Components of aggressive end-of-life care**			
new chemo in last 30 days		5 (35.7%)^a^	
any chemo last 14 days		3 (21.4%)^a^	
intensive care unit		1 (7.1%)^a^	

a Choosing multiple responses possible.

### Differences in treatment decisions depending on the degree of aggressive care

According to Simon’s distinction between the process of deciding and the process of action upon these decisions, we identified a similar distinction in our material between decision-making (how decisions unfolded and the process of negotiating a treatment choice) and decision-taking (how people actively committed to decisions and acted upon it). In our study, depending on whether the patient received aggressive end-of-life care or not, the extent to which third parties were involved differed markedly. While patients in the aggressive care group seemed to prefer making *and* taking treatment decisions collectively with their family members and/or their physicians, patients who did not receive aggressive end-of-life care tended to also prefer a collective decision-*making*, however, eventually *took* the decision alone.

Patients never made the decisions concerning aggressive end-of-life care themselves, but at the very least, sought the advice of either their attending physicians, family members, or both parties. By analyzing the aggressive care group more thoroughly, we found that there was a particularly strong involvement of family members in the decision-making process, meaning that all treatment options, risks, and likely consequences were closely discussed between the patients and their family members. This was reflected in the fact that medical appointments were attended together, and family members joined the discussion about treatment options between patients and physicians, as displayed in the following quote:

Participant #8: *“Therefore, it was my mother and I [who were responsible for the decision upon a treatment]. Actually, it was mainly me who talked to the doctors in the NCT or wherever.”*

Interviewer: *“That means you were always there?”*

Participant #8: *“Yes, we always were a team.”* [Participant #8]

However, most of the time the patients and the family members would take their time to discuss and assess different options together based on and guided by the doctors’ advice:

“*And therefore, we decided to take this path. It was a decision between us both – equally. [ … ] We discussed it openly and could talk in a factual manner with the doctors. Getting things explained. Therefore, he was able to take a decision and to discuss it.”* [Participant #16]

Almost all interviewed family members put a strong emphasis on the fact that they had always been with the patient, either physically or emotionally or both. This was apparent by the common use of the first-person voice, since “we”, “us” or “together” was, in contrast to the non-aggressive care group, regularly used when they talked about decisions. Similarly, the moment of deciding upon a treatment modality was, according to most interviewees, a natural result of a joint discussion process and, within the aggressive care group, often initiated or at least demanded by the patient’s family members:

Participant #14-01: “*I always said: [name of patient] fight. Let’s fight together, we will do this. And then he said, someday he said to me, I don’t want, I use his words, this damn chemo. This is my end. And then the chemo eventually started since nothing else would work.”*

Participant #14-02: “*Since it [the tumor] progressed fast and the chemo promised a little bit more time.”*

Interviewer: *“Okay. So, it was clear for you two that it was going downhill, and that the chemo would only mean a prolongation [of survival time]? Even if a recovery was out of sight, at least a little bit more time trough chemo?”*

Participant #14-02: “*Exactly.”* [Participant #14]

The above quote also covers one of the main reasons for choosing aggressive end-of-life care, that is the opportunity to spend more time with the loved ones. In almost all cases, a cure was deemed impossible, so the decision to use aggressive end-of-life care would only lead to an extension of life. Some family members even explicitly reasoned the patients into choosing the aggressive treatment, while others reported that it was important for them to have the patient near them, even if the caretaking meant great effort. Another reason for choosing aggressive end-of-life care was the patients’ unwillingness to accept the fatal diagnosis as they struggled not only to survive longer but to cure the cancer. Nevertheless, taking this path often led to a hurtful dynamic between the patients and the family members as shown in one case, where the patient’s clinging to the idea of a curative treatment approach resulted in a profound detachment between the patient and the desperate family members:

“*When we sat in the kitchen crying after receiving bad news, and there were plenty, he was angry: “Why are you crying? What’s up with you? Everything is fine. Why do you always make such a fuss?”* [Participant #12]

Most patients, however, took a more pragmatic stance and endured the sometimes painful and debilitating procedures related to aggressive end-of-life care in hope of being able to spend some more time with their loved ones:

Interviewer: “[ … ]*, buying time and you said, this was primarily for you two to have more time, to not be left alone?”*

Participant #14-02: “*If it wasn’t for her [the patient’s wife] he would have decided completely different. Definitely. Am I allowed to say this?”* [Participant #14]

Comparing the descriptions of the both groups in the entire body of the interview data, the process of how decisions were made was similar. However, the description of the process of how decisions were *taken* differed markedly between these two groups. While the aggressive care group had ongoing family involvement of family members in both steps of negotiating treatment decisions, patients in the non-aggressive care group ultimately took the decision upon a treatment on their own. In this group, the role of the family members was confined to gather information about treatment options and to participate in preliminary discussions about potential ways to proceed. In some cases, patients excluded family from the decision-making process even at this early stage and turned exclusively to their treating physicians. While this early involvement of family members or physicians prepared the final decision to some extent, patients in the non-aggressive care group regularly took the final decision upon a treatment alone.

Given the discrepancy concerning decision-taking between the groups, we further investigated potential explanations for the specific nature of how decisions against aggressive end-of-life care evolved.

### How did patients eventually *take* decisions against aggressive care?

Overall, decision making was negotiated between patients and family members, but patients eventually *took* the decisions against aggressive care on their own, that is at a certain point, they stopped involving their partners, their family members, or their physicians. In a next step, we therefore investigated *how* patients actually implemented the decision against aggressive end-of-life care and identified two key factors, namely physical exhaustion, and precautionary protecting of family members from feelings of guilt.

First, many patients did not act upon their decision against aggressive care until they felt extremely weak or even physically exhausted. It turned out that the bodily experience of rapidly dwindling physical strength, made the last step of implementing the decision easier for those affected. Sometimes the physical decline was due to the malignant disease itself, sometimes to massive adverse effects of the therapy (mostly chemotherapy):

*“In the end, it was a bit like that, that she - if I understood it correctly - basically wanted us daughters to take care that she was allowed to die, if certain things were like that and so they were. [ … ] with my mother I could see it very clearly [ … ] She could even communicate a little bit, very quietly. It just worked out so that I understood it. However, she could no longer eat, and she could no longer hold her bowel movements. She said, ‘There’s no point now’, and I understood that quite well. I could understand that very well, that boundary.”* [Participant #13]

In the course of the patients taking their own poor physical condition as a reason to implement their decision against aggressive end-of-life care, it was often made easier for family members to cope with this difficult decision. In most cases, they had observed the deterioration of the patient’s physical condition over a long period of time and with a certain feeling of helplessness. Moreover, the implementation of the decision gave the family members hope that they could spend the little time they had left together free from the restrictions caused by the adverse effects of chemotherapy:

Interviewer: *“When your wife decided not to have another chemotherapy, how did you feel about it? Did you have more of an impulse that it would be good to have another one after all?”*

Participant #11: *“No. [ … ] I saw how it took a lot out of her, so I said, if that’s what she wants, then I’ll accept it.”*

Interviewer: *“Are you referring to the various adverse effects?”*

Participant #11: *“Yes, my mind said I can understand that – it always hurts.”*

Interviewer: *“Emotionally, would it have felt good to try one more treatment?”*

Participant #11: *“If there had been a chance. But the metastases had grown firmly, despite chemo. I believed that it would only be delayed for a relatively short time and in this situation is better to do without the adverse effects.”* [Participant #11]

In contrast, if patients tolerated chemotherapy well, they and their family members regarded chemotherapy to be without an alternative given the threat posed by the cancer disease:

*“There appeared to be no alternative, since, if we didn’t do this chemotherapy, the cancer would have won in the first place. And that’s why we decided for the chemotherapy. And during the second cycle, the drug was switched, so not this Cisplatin, but Carboplatin. And that one my wife tolerated much better.”* [Participant #4]

Moreover, the experience that the chemotherapy was entirely, or at least largely, free of adverse effects seemed to relieve the pressure on participants to make a more fundamental decision regarding the scope of treatment. Rather, only the prolonged experience that the chemotherapy did not work seemed to enable a fundamental decision on the further use of the therapy:

*“Would we have done a fifth cycle or a sixth cycle? I must tell you; I don’t know. We would have decided cycle by cycle. But on the other hand, at least in theory, the more advanced the disease is and the more unsuccessful the chemo is, I believe, then at a certain point you will almost automatically say, let’s see if we continue.”* [Participant #4]

A second important factor for taking the decision against aggressive care alone was that patients did not want to run the risk of feelings of guilt arising or remaining with their family members after the death. Hence, they chose to implement the decision themselves ending a grueling rollercoaster of emotions, namely between hope and resignation, for all parties involved. In this way, at least in the medium term, patients also took the responsibility away from the family members and relieved them of the tremendous emotional burden related to this decision:

*“I have seen it with other people: When people maintain hope, then there is this up and down. There is hoping and being disappointed again and then you give it [chemotherapy] another go when you are told “we have found a new drug”. All of this was now overcome, so to speak. In this regard, she [the wife] had acted upon the decision saying “I will do this now. it can come as it comes, but I don’t have to go to chemo now.”* [Participant #2]

Interviewer: *“You always went with him. Were you also present in the discussions with the doctors?”*

Participant #6: *“No, he always didn’t let me go with him.”*

Interviewer: *“Sometimes he wanted to discuss it alone?”*

Participant #6: *“Yes, apparently, he didn’t want me to know everything. I do not know…*

Interviewer: *“Do you have the feeling that your husband wanted to protect you a bit, that you should not hear all the words?”*

Participant #6: *“Yes, he was so grateful for everything. He once said to my son that he had never known what he had in me.”* [Participant #6]

In this process of clarifying the assumption of responsibility for the decision taking, the physicians informed about the disease and treatment options, but often also took on a kind of moderator role. In this role, they spoke directly to the patients to ideally receive a response that would be less colored by loyalties to their important other:

*“Then she [the oncologist] said, let your wife talk. [ … ] I also thought that was correct, I really took a step back there. “I would like to hear this from your wife”, she [the oncologist] said. I think that is correct, so that it’s not so wishy-washy that my wife is doing it for my sake, right? That’s what she [the oncologist] wanted to hear from my wife’s mouth.”*

[Participant #2]

All in all, patients and their family members seemed to be trapped in a cycle between optimism and resignation from the moment of diagnosis. Decisions for chemotherapy at the end of life and the adherence to these decisions were almost automatic, unless adverse effects or massive physical weakness occurred. While patients and their family members, supported by the treating physicians, then discussed the possible further viable paths, the ultimate responsibility for the decision against aggressive therapy remained with the patients. In some cases, the data suggested that the patients wanted to protect their family members from additional stress and later feelings of guilt that they, the family members, might have done something wrong.

## Discussion

### Principal findings

In this study, we employed a qualitative approach to thoroughly investigate the interplay between patients, their family members, and physicians when dealing with treatment decisions in the context of aggressive care at the end of life. Moreover, we focused on situations when decisions against aggressive care were taken. First, we identified a difference in the decision process between the aggressive care and the non-aggressive care group: Patients of the former group tended to make *and* take decisions with their family members and physicians; the decision was negotiated until it was finally implemented. In contrast, patients of the non-aggressive care group did discuss important treatment decisions with their family members and physicians, but eventually *took* the decision against further treatment alone. Second, the main reason for deciding in favor of aggressive end-of-life care seemed to be a desire to spend some more time with loved ones, and sometimes a strong will to live on. Lastly, a decision against aggressive end-of-life care, usually taken by the patient alone, had mainly two motives. The first was rapid decline of health and general physical exhaustion due to the adverse effects of intense therapy (especially chemotherapy) or the progressive cancer, which led to the decision against any further therapy to potentially preserve the remaining quality of life. The second motive for deciding against aggressive end-of-life care was the wish to keep loved ones away from emerging feelings of guilt and self-accusation for seemingly “wrong” decisions, which led to the exclusion of loved ones from the decision-taking process.

### Limitations

This study has several limitations. First, the interviews took place on average two years after the death of the patient. Between both groups (aggressive end-of-life care vs. non-aggressive end-of-life care), the mean period between death of the patient and interview participation differed by ten months. Given this difference and the fact that family members described the circumstances of the loss of a loved one, our data may be not only susceptible to recall bias but also the risk of bias may be different between the two groups. A much more detailed and reliable description and assessment of the treatment decisions by additionally interviewing the patients themselves during the process of decision making and decision taking will be possible in future cohort studies. In the present study, for ethical reasons, we refrained from contacting the bereaved caregivers earlier than six months after the patient’s death for participation in the initial survey. Due to logistical circumstances (e.g., arranging appointments, conducting the survey, exploring interview interest, arranging and conducting the interview), we were unable to conduct interviews in a timely manner in some cases.

Second, the interviews were conducted in 2015 and 2016 which is a relatively long time considering the fast development in cancer treatment research. That may have led to the fact, that the circumstances in which the patients and the caregivers are confronted with difficult treatment decisions have changed since when we conducted the interviews. However, publications from the past years analyzing newer data reported comparable findings and difficulties from interviews with caregivers, patients and physicians regarding decision-making processes at the end of life (23–25).

Third, our sample of bereaved family members consisted of spouses (n=11) and children (n=5) of the deceased cancer patients. Consequently, our findings on family members’ experiences with end-of-life care in general and aggressive end-of-life care in particular are limited to these two groups. Furthermore, the proportion of spouses and children differed between the two groups with two-thirds being the child of the deceased in the aggressive care group and 90% being the partner of the deceased in the other group. This could also explain the large age difference between the two groups of interviewees. It may also contribute to the finding, that patients who choose not to receive against aggressive end-of-life care alone may want to protect their loved ones from feelings of guilt, since this obligation may be even stronger for patients if the loved one to be protected is their own child. Future studies may particularly focus on this aspect and also include siblings or parents, a strategy that, however, comes with its own specific challenges. Even the survey sample in our project included very few siblings and parents of deceased cancer patients (18). Fourth, there were differences between the both groups regarding age in our sample. The median age of patients who experienced aggressive end-of-life care was 12,5 years lower than of those who did not. In general, a higher age is associated with less experiencing of aggressive end-of-life care in advanced stage cancer patients (4, 26–30). The reason for this could be the prognosis that younger patients tolerate the side effects of aggressive treatment better than older patients. However, oncologists often struggle with the correct prognosis of survival and performance status and overestimate them which consequently lead to aggressive care (1, 31). However, in our own quantitative study in which we included 298 family members of deceased cancer patients, there was no difference between the two groups regarding age (18). In that study, we found that family members of patients who had experienced aggressive end-of-life care suffered from significantly higher decision regret compared to family members who had not experienced aggressive end-of-life care.

Fifth, we could not provide data on physicians’ treatment recommendations and how they assessed the individual situation. This would have been helpful to gain a holistic view on the circumstances in which the treatment decisions were made and taken. However, in the interviews the caregivers often mentioned the physicians’ role and their influence regarding the treatment decision-making process.

Finally, in a few cases, assignment to the respective group was not clear-cut. The information given by the interviewees somewhat contradicted the group affiliation that was determined based on the answers given in the preceding survey. Given the relatively strict data protection regulations in place in the German health care system, there is currently no nationwide cancer registry from which we could have extracted information about the course of disease and service provision at the end of life for the respective advanced-stage cancer patient. Hence, we cannot rule out that family members may have remembered the cancer treatment trajectory vaguely or even incompletely. Nevertheless, there is ample evidence that proxies do reliably report service provision at the end of life (32).

### Comparison with prior work and contributions beyond

Our findings on treatment decision processes including the interplay between patients, family members, and physicians regarding advanced-stage cancer care are mostly in line with other reports. A recently published qualitative study from the Netherlands also investigated the process of decisions by focusing on the involvement of patients, family members and physicians (23). Participants mentioned quality of life and burden from the treatment as reasons for stopping treatment and the will to live longer and not to die as reasons for prolonging treatment. Our findings add insights into the differential role of family members depending on the way the decision-making process took.

A group from Germany investigated the roles of patients, family members, and physicians in treatment decision processes. In several studies, the group investigated how these different stakeholders contribute to the final decision on cancer treatment. In line with the conceptual approach of differentiating between “decision-making” and “decision-taking” as applied in our study, the German group reports from a cohort study that two-thirds of the family members acted as advocates for the patients’ preferences, but only one-third were actually involved in the treatment decision process. Another finding was that disagreements about which treatment option to take often ended with choosing aggressive care – which was the modality preferred by the family members (33). Family members in our study rarely mentioned disagreements between patients and themselves. The mutual discussions about treatment options were described mostly as constructive. It seems plausible, that by taking the decision against aggressive care on their own the patients not only protected their family members from feelings of guilt but also prevented broad discussions on potentially emerging disagreements. As we only conducted interviews with the bereaved family members, we cannot describe (1) how patients experienced possible discussions with family members before taking the decision and (2) what their subjective motives were for taking the decision not to receive aggressive care alone. Due to the retro perspective design of our study, we were unable to validate the caregivers’ reports with the perspective of the deceased patients.

Regarding the involvement of cancer patients in treatment decisions, in a sample of 70 patients it was estimated that only 55% were informed about life-prolonging treatment options given their specific cancer diagnosis and the course of treatment by their physicians. Only 73% of the physicians were aware of the patients’ wishes and treatment preferences (34). These observations demonstrate the importance of involving family members in important treatment decisions and enable patients to communicate their wishes to their physicians. Physicians should be enabled to recognize the important role of family members and involve them early in the decision-making process, ideally based on a systematic assessment of family members’ needs and wishes (35). In an interview study from the US, the authors report that disagreements between patients with advanced lung cancer and their family members about treatment decisions are common and do cause problems in the communication and consequently in the decision process. To address these challenges, communication with the families should be sought focusing on patients’ and family members’ satisfaction with treatment decisions (36).

Overall, treatment decisions about starting, continuing, or discontinuing potentially life-prolonging treatment at the end-of-life are always individual. Therefore, perceptions regarding disease status and treatment options at a given time may differ between patients and family members. Perceptions about ‘appropriate’ or ‘inappropriate’ care in the last phase of life are known to differ between the parties involved (37). While aspects such as being well informed about treatment options and following the patient’s wishes are consistently seen as appropriate care, the decision for potentially curative or life-prolonging treatment are often deemed as both inappropriate and appropriate care. Appropriate in that it may offer hope to patients and their family members, a chance to prolong life, or it simply be in accordance with the patient’s wishes. However, in many cases, aggressive cancer care may also induce false expectations, adverse events, and complications such as severely diminished quality of life. The findings of our study reflect that family members may assess similar situations and treatment decisions very differently, depending on the individual perceptions and experiences or how the physicians communicate with the different stakeholders.

## Conclusion

Family members and doctors of patients with advanced cancer are regularly involved in the decision-making process concerning aggressive care. Notwithstanding, our study also shows that patients who decide against aggressive care implement their decision without further involvement of their family members. The main motive is to protect the relatives from feelings of guilt and self-accusation for seemingly “wrong” decisions. For clinicians, these findings imply to include the family members and patients in the decision-making process and revisit them repeatedly to be aware of their preferences. By a profound understanding of their needs and wishes, medical personnel can facilitate the decision process as well as moderate in the case of conflicting interests. At any rate, clinicians should validate patients and family members who consider refraining from aggressive care and explore their motives.

To account for a longitudinal perspective on the interplay between patients, family members, and physicians negotiating decisions on aggressive end-of-life care, cohort studies are needed. Cohort studies will enable the prospective and systematic assessment of (1) the individual perceptions of patients, family members, and physicians on aggressive care during the course of disease, (2) the actual extent to which the parties are involved in the decision-making process, and (3) critical timepoints for tailored interventions fostering both communication and conflict resolution between patients and family members and the prevention of decision regret and feelings of guilt in family members.

## Data availability statement

The original contributions presented in the study are included in the article/**Supplementary Material**. Further inquiries can be directed to the corresponding author.

## Ethics statement

The studies involving human participants were reviewed and approved by Ethics Committee of the Medical Faculty at Heidelberg University (S-500/2014). The patients/participants provided their written informed consent to participate in this study.

## Author contributions

MWH, MH, DJ, and H-CF conceptualized the study. CB, NB, JT, and MWH collected, prepared, analyzed, and visualized the data. MWH, JT, MH, DJ, CB, and H-CF drafted the manuscript. All authors revised the manuscript critically for important intellectual content. All authors contributed to the article and approved the submitted version.

## Funding

Financial support for this study was provided entirely by a grant from the German Research Foundation (Deutsche Forschungsgemeinschaft, DFG) (Grant no. HA 7536/1-1). The funder was not actively involved in conduct of the study. The authors had full access to all of the data in this study and take complete responsibility for the integrity of the data and the accuracy of the data analysis.

## Acknowledgments

We would like to thank Linda Söderberg, MSc (Psych), for assisting in the data collection.

## Conflict of interest

The authors declare that the research was conducted in the absence of any commercial or financial relationships that could be construed as a potential conflict of interest.

## Publisher’s note

All claims expressed in this article are solely those of the authors and do not necessarily represent those of their affiliated organizations, or those of the publisher, the editors and the reviewers. Any product that may be evaluated in this article, or claim that may be made by its manufacturer, is not guaranteed or endorsed by the publisher.

## Abbreviations

NCT, National Center for Tumor Diseases.
