# Supplementary material for: Negotiating decisions on aggressive cancer care at end-of-life between patients, family members, and physicians – A qualitative interview study
Source: Front Oncol. 2022 Sep 23;12:870431. doi: 10.3389/fonc.2022.870431 (PMC9539079; doi:10.3389/fonc.2022.870431)
Supplement: Supplementary file 1 [file DataSheet_1.docx]

**SUPPLEMENTAL APPENDIX**

**APPENDIX 1 – SEARCH TERMS FOR MEDLINE SEARCH**

*Last search date: February 2^nd^, 2022, no filters applied, 1,106 results*

(“Family members”[Title/Abstract] OR “Family member”[Title/Abstract] OR caregiv*[Title/Abstract] OR “significant other”[Title/Abstract] OR “significant others”[Title/Abstract] OR “proxy”[Title/Abstract] OR “proxies” [Title/Abstract]) AND ((prolong* OR aggress*[Title/Abstract]) AND (care[Title/Abstract] OR treatment [Title/Abstract] OR end-of-life care[Title/Abstract] OR therapy[Title/Abstract])) AND (qualitative[Title/Abstract] OR interview[Title/Abstract] OR perspective*[Title/Abstract] OR group*[Title/Abstract])

**APPENDIX 2 – SEMI-STRUCTURED INTERVIEW GUIDE**

Description of the treatment and decision-making process (narrative perspective):

- First, I would ask you to describe your deceased relative and what kind of person he/she was.
- Would you please tell me the life course and the progression of disease of your deceased relative during the last six months before death in detail?
- What was the treatment process during the last six months before death like? What kind of treatment (chemotherapy, radiotherapy, operation) did your relative receive during this period of time?
- How was the individual decisions process concerning further therapy? In which way was your relative involved in the decision making process? What was the role of the attending physicians in this process?
- What was your part in the decision-making process as a relative? Were you included? If yes, in what way?
- If you were included in the decision-making process concerning further therapy, what was your stance? Where there helpful or unfavorable circumstances that influenced the decision-making process?
- With whom did you speak – inside hospital or outside of hospital (e.g. physicians, relatives, and friends)?
- On what basis did your deceased relative make his/her decisions? Where there helpful or unfavorable circumstances that influenced his/her decision-making process?
- With whom (inside and outside of hospital) did your deceased relative talk about the upcoming decisions?
- Have there been any conflicts in negotiating further treatments? If so, between whom did they occur? How did the involved persons deal with them?
- Have you experienced situations, in which you did not share your relatives’ perception of further treatment? If yes, have you been able to discuss these differences with someone? If yes, with whom did you talk?

Evaluation of the therapeutic and decision-making process (identification of perceptions):

- Looking back on the last four weeks of your deceased relative’s life, how do you feel?
- Do you feel like having been able to participate in the therapeutic process to a satisfying extent?
- From your point of view, how satisfied was your deceased relative with the therapeutic decisions and the therapeutic process in the last months before his/her death?
- Have there been any developments which you regret from your present point of view? If yes, how would you act differently, or advise other people, which are in the same position?
- Did you or your deceased relative have the need to talk about the subject of death and dying in the last couple of weeks?
- Did you have the feeling there was enough time and space to talk about death and dying, as well as to say goodbye? If yes, what contributed to this situation? If not, what prevented this from happening?
- From the present point of view, do you still feel burdened with the experiences concerning the end of life of your deceased relative? Does it affect you in your daily life? If not, what helped you to cope with the experience?

Concluding questions:

- At the end, I still have some specific questions: in which environment did your relative die?
- Is there anything left to talk about, which is important to you?

Thank you for the interview.

**APPENDIX 3 – SUMMARY OF KEY THEMES**

| **Key theme** | **Definition** | **Subthemes** | **Supporting quotes** |
| --- | --- | --- | --- |

| **Actors involved in the decision-making process** | Actors involved in the discussion of different treatment or non-treatment options, getting further information on treatment or non-treatment options and assessing consequences of treatment or non-treatment options. | patient | “Maybe that [opinion of relatives] influenced him, but I think it mainly influenced him in the way he expressed himself, not in the therapy decision. I think that was already the case with him. He really fought and that's what he wanted himself, yes.” [Participant #1] |
| --- | --- | --- | --- |
|  |  | patient and relative | “And then he would say: ‘Should we do that?’ And he would always get the opinion of Paula [daughter] and me and then say: ‘Okay, this and that’. And that was usually the right thing to do. We reached a consensus.” [Participant #7] |
|  |  | patient and physician | “He was very fond of research and science. Simply because he was a professor himself. It's true that he was in a completely different field, business administration, but he had a lot of trust in the doctors or what they told him. He read up on a lot of things, but when he was told that something could be done that could be explained to him conclusively, it was such a mixture of information and hope that he decided in favor of it.” [Participant #16] |
|  |  | all parties | “Let's say three parties. First of all, the doctor from the thoracic clinic who was in charge of the therapies. The pneumologist was the coordinator and my wife, as the person affected, was the decision maker whether yes or no.” [Participant #4] |

| **Actors involved in the decision-taking process** | Actors involved in the specific moment the decision was taken. | patient | “Yes, that's how it started, she took care of herself and read up on things and informed herself. She did all that herself. So, she decided very much on her own. Even that she wanted to have the operation in the first place and also afterwards that she wanted to have chemo, she did all that herself.” [Participant #13] |
| --- | --- | --- | --- |
|  |  | patient and relative | “[...] but in the end we then said that he should do it [ reference to intestinal surgery] so that we are on the safe side.” [Participant #5] |
|  |  | patient and physician | “He decided that on his own. If the doctors told him to do it, he did it. He wanted to get better.” [Participant #6] |
